# Effect of highly branched hyphal morphology on the enhanced production of cellulase in *Trichoderma reesei* DES-15

**DOI:** 10.1007/s13205-016-0516-5

**Published:** 2016-10-05

**Authors:** Ronglin He, Chen Li, Lijuan Ma, Dongyuan Zhang, Shulin Chen

**Affiliations:** 1 Tianjin Institute of Industrial Biotechnology, Chinese Academy of Sciences, Tianjin, 300308 People’s Republic of China; 2 Key Laboratory of Industrial Fermentation Microbiology, Ministry of Education, Tianjin University of Science and Technology, Tianjin, 300457 People’s Republic of China; 3 Tianjin Key Lab of Industrial Microbiology, Tianjin University of Science and Technology, Tianjin, 300457 People’s Republic of China; 4 College of Biotechnology, Tianjin University of Science and Technology, Tianjin, 300457 People’s Republic of China

**Keywords:** Hyphal morphology, *Trichoderma reesei*, Cellulase production, Branching, Submerged cultures

## Abstract

The morphology of *Trichoderma reesei* is a vitally important factor for cellulase productivity. This study investigated the effect of hyphal morphology on cellulase production in the hyper-cellulolytic mutant, *T. reesei* DES-15. With a distinct morphology, *T. reesei* DES-15 was obtained through Diethyl sulfite (DES) mutagenesis. The hyphal morphology of DES-15 batch-cultured in a 5-L fermentor was significantly shorter and more branched than the parental strain RUT C30. The cellulase production of DES-15 during batch fermentation was 66 % greater than that of RUT C30 when cultured the same conditions. DES-15 secreted nearly 50 % more protein than RUT C30. The gene expression level of a set of genes (*cla4*, *spa2*, *ras2*, *ras1*, *rhoA*, *cdc42,* and *racA*) known to be involved in hyphae growth and hyphal branching was measured by quantitative real-time PCR. The transcriptional analysis of these genes demonstrated that a decrease in gene expressions might contribute to the increased hyphal branching seen in DES-15. These results indicated that the highly branchin*g* hyphae in DES-15 resulted in increased cellulase production, suggesting that DES-15 may be a good candidate for use in the large-scale production of cellulase.

**Electronic supplementary material:**

The online version of this article (doi:10.1007/s13205-016-0516-5) contains supplementary material, which is available to authorized users.

## Introduction

Much research has been performed with the aim of developing renewable energy sources as alternatives to fossil fuels. The high cost of lignocellulosic enzymes for lignocellulosic degradation is one of the most important limiting factors in the bio-refinery of lignocellulosic biomass (Wilson 2009). The bio-refinery industry currently uses lignocellulosic enzymes that are primarily produced by ascomycete fungi, such as species of *Trichoderma*, *Penicillium*, or *Aspergillus* (Gusakov 2011). The enzymes are secreted into fermentation broths at high levels following submerged cultivation. Submerged cultivations permit the easy acquisition of secreted enzymes for downstream fermentation processes (Liu et al. 2013). Submerged cultures are generally employed for the large-scale production of filamentous fungi. Widely recognized as complex systems, submerged cultures could be influenced by many external factors, some of which can affect the hyphal morphology of filamentous fungi (Kaup et al. 2008). In most filamentous fungal fermentation, it is generally believed that the level of productivity is dependent on the ability of the fungus to achieve an optimal morphology (Krull et al. 2013). In *Aspergillus niger*, increasing the stirring speed and adjusting the glucose concentration promotes citric acid production, thereby resulting in changes to the fungal morphology (Papagianni et al. 1998, 1999; Amanullah et al. 2002). The relationship between the external environment and fungal morphology has been comprehensively investigated as a means to optimize fungal morphology. Numerous studies have demonstrated that changing important parameters during the fermentation process, such as the initial spore concentration, medium composition, pH value, temperature, and agitation, can control fungal morphology (Gibbs et al. 2000; Papagianni 2004; Ferreira et al. 2009; Peciulyte et al. 2014).

*Trichoderma reesei* have been widely used in a broad range of industrial applications, and have become the main source of lignocellulosic enzymes (Dashtban et al. 2009). Typically, when using submerged cultures for the large-scale production of cellulase, any alteration to the hyphal morphology of *T. reesei* can greatly influence cellulase yields (Domingues et al. 2000). It is generally believed that protein secretion by filamentous fungi primarily occurs at the young hyphal tips (Gordon et al. 2000), where the cell wall is more porous and facilitates rapid protein secretion (Chang and Trevithick 1972). The process of branching can lead to the emergence of more growing tips that are important for protein secretion (Peberdy 1994). Any internal or external factors that can increase the number of active tips have the potential to improve the overall protein yield (Juge et al. 1998). For example, Ahamed and Vermette (2009, 2010) reported that the number of hyphal branching and hyphae tips could be controlled through manipulating agitation rates and culture medium composition, a method that could be employed to help improve cellulase activity. However, the previous studies were focused on manipulating only a single parameter, due to the extreme difficulty associated with simultaneously adjusting all influencing factors during fermentation. Nevertheless, obtaining a strain with optimal morphology in submerged cultures is an important factor for the production of cellulase by *T. reesei*.

In this study, we utilized a hyper-cellulolytic mutant (DES-15) with a unique morphology, where the hyphae appeared to be shorter and more branched during fermentation. DES-15 was originally obtained by dimethyl sulfate (DES) mutagenesis. During the fermentation process, cellulase productivity and protein secretion were significantly improved in the DES-15 mutant when compared to the RUT C30 strain. Furthermore, to elucidate the relationship between hyphal morphology and cellulase productivity, we preformed a transcriptional analysis on DES-15 to determine the genes involved in hyphal branching during the submerged culture process.

## Materials and methods

### Fungal strains, media, and cultivation conditions

*Trichoderma reesei* RUT C30 (ATCC 56,765) was used as the parent strain all throughout this study. The hyper-producing cellulase mutant DES-15 was generated from the parent RUT C30 strain using diethyl sulfite (DES) mutagenesis, and was utilized throughout. All strains were maintained on potato dextrose agar (PDA) medium at 4 °C. For recovery of strains, mycelia agar disks were inoculated on fresh PDA and cultivated at 30 °C for 7 days until conidia formed.

The inoculum medium was composed of 2 % (w/v) avicel cellulose, 1.7 % (w/v) corn steep liquor, and 0.2 % (w/v) glucose. The pH was adjusted to 4.5 using 2 M of H_2_SO_4_.

The fermentation medium contained (w/v) 3.3 % avicel cellulose, 1.7 % corn steep liquor, 0.5 % (NH_4_)_2_SO_4_, 0.6 % KH_2_PO_4_, 0.1 % MgSO_4_·7H_2_O, 0.25 % CaCO_3_, 0.25 % glycerol, and 0.1 % Tween-80. Initially, the pH of the fermentation medium was adjusted to 5.5 using 2 M H_2_SO_4_.

### Mutagenesis and screening of the cellulase hyper-producing mutants

DES mutagenesis was performed as follows: 4 mL of the spore suspension (10^7^ spores/mL) was treated with 0.2 mL of DES in 16 mL of sterile 0.1 mol/L potassium phosphate buffer (pH 7.0) at 30 °C for 40 min and mixed at 150 rpm with constant shaking. The treated spores were then spread onto PDA and incubated at 30 °C for 3–5 days until colonies appeared. Single colonies were picked and seeded onto fresh PDA plates for purification, and inoculated into the fermentation medium for enzyme activity analysis.

### Enzyme production in shake flasks and in a laboratory-scale fermentor

Approximately 1 mL of spore suspension (10^7^ spores/mL) was inoculated into 250 mL Erlenmeyer flasks containing 30 mL of inoculum medium, and incubated with constant shaking (200 rpm) for 24 h at 30 °C. A total of 1.5 mL of pre-culture media was then inoculated into 250 mL Erlenmeyer flasks containing 30 mL of the fermentation medium (Li et al. 2013). The Erlenmeyer flasks were incubated with rotary shaking (200 rpm) at 26 °C for 120 h.

Batch cultivation was carried out in a 5–l laboratory-scale fermentor (BIOTECH-5BG, Shanghai Baoxing Bio-Engineering Equipment Co. Ltd., China). The cultivation parameters were as follows: pressure 0.05 Mpa, pH 5.0, and temperature 26 °C. The dissolved oxygen (DO) was controlled at 30 % by varying the agitation speed and airflow rate. 10 % (v/v) silicone antifoaming agent (THIX 298, Yantai Hengxin Chemical Co., Ltd., China) was automatically added into the fermentor by the fermentor controlling system for foam controlling. 2 M ammonia or 2 M sulfuric acid was automatically added into the fermentor by the fermentor controlling system as needed to carefully maintain the culture pH. The bioreactor’s working volume was 3-L, from which samples were periodically withdrawn, centrifuged at 6000 *g* for 5 min, and the enzymatic activities determined from the collected supernatants. These experiments were conducted separately three times.

### Protein, dry cell weight (DCW) of mycelia, and enzyme assays

For extracellular protein determination, the fermentation broth was centrifuged at 8000 rpm for 10 min and the supernatants were used for extracellular protein determination. The extracellular protein concentration was determined using a standard Bradford assay, as has been previously described (Bradford 1976).

The dry cell weight (DCW) of mycelia was measured using the perchloric acid method (Ma et al. 2013). By this method, the mycelia weight was calculated from the difference between the total dry weight of the solid (mixture of mycelia and cellulose), and that of the residual cellulose. The dry weight of the solids was determined as follows: 20 mL of the cultures were centrifuged at 10,000 rpm for 20 min; then, the pellets were washed three times with 10 mL of water, and finally lyophilized until a constant weight was obtained.

The FPase activity was measured as described by Ghose (1987). Briefly, this method measures the release of the reducing sugars produced. The amount of reducing sugars produced was determined following a 60 min culture, where 500 μL of an enzyme solution is mixed with 1 mL of citrate buffer (0.05 M, pH 4.8) in the presence of 50 mg of Whatman No. 1 filter paper, and incubated at 50 °C. The FPase activity was represented by the filter paper unit (FPU), which was defined as the amount of enzyme required to produce 1 μmol of reducing sugar in 1 min. The cellulase production for each strain was represented by its FPase activity in the fermentation broth.

### Microscopy

To examine the hyphal morphology, 10 μL of the mycelia samples was diluted in 1000 μL of distilled H_2_O, and 10 μL of the diluted sample was placed onto a microscope slide. The slides (after heat fixation) were then observed and recorded using a CX-31, Olympus microscope. In each sample, at least 3000 hyphae were examined from 30 random bright fields. The single hypha length, hypha diameter, numbers of tips, and numbers of branches were measured using the IPP software (Image-Pro Plus 6, Media Cybernetics).

### RNA isolation and quantitative real-time PCR

The fungal mycelia were harvested by filtration, washed with distilled water, frozen, and then ground under liquid nitrogen. Total RNA was isolated using the Trizol extraction method (Life Technologies, USA). cDNA synthesis was performed using a RevertAid™ H Minus First Strand cDNA Synthesis Kit (Fermentas, MA) using the oligo dT primers which were provided in the kit. To ensure the validity of our findings, we evaluated the RNA of each strain by Reverse Transcription PCR (RT-PCR) to verify that no genomic DNA was carried over in the samples during the experimental process.

Quantitative real-time PCR (qPCR) assays were performed in an Applied Biosystems 7500 Fast Real-Time PCR System (Invitrogen™ Life Technologies, USA) according to the manufacturer’s instructions. Each reaction mixture contained 1 μL of the template cDNA (10 ng/μL), 10 μL of SYBR premix (Takara, Japan), 0.2 μL of both the forward and reverse PCR primers (10 μM), and the volume of nuclease-free water required for a final volume of 20 μL. The PCR program is described as follows: first, there was an initial denaturation at 95 °C for 30 s, followed by 3 s at 95 °C and 30 s at 60 °C (40 cycles). A melting curve was performed after each run to check the specificity of the PCR product. The qRT-PCR primers used are listed in Table S1. The samples were analyzed in at least two independent experiments with three replicates in each run. The transcript levels of the target genes were normalized against the transcript levels for the *gpd1* gene using the 2^−ΔΔCt^ method as has been previously described (Livak and Schmittgen 2001).

## Results and discussion

### Screening of hyper-producing cellulase mutants

**Fig. 1:**
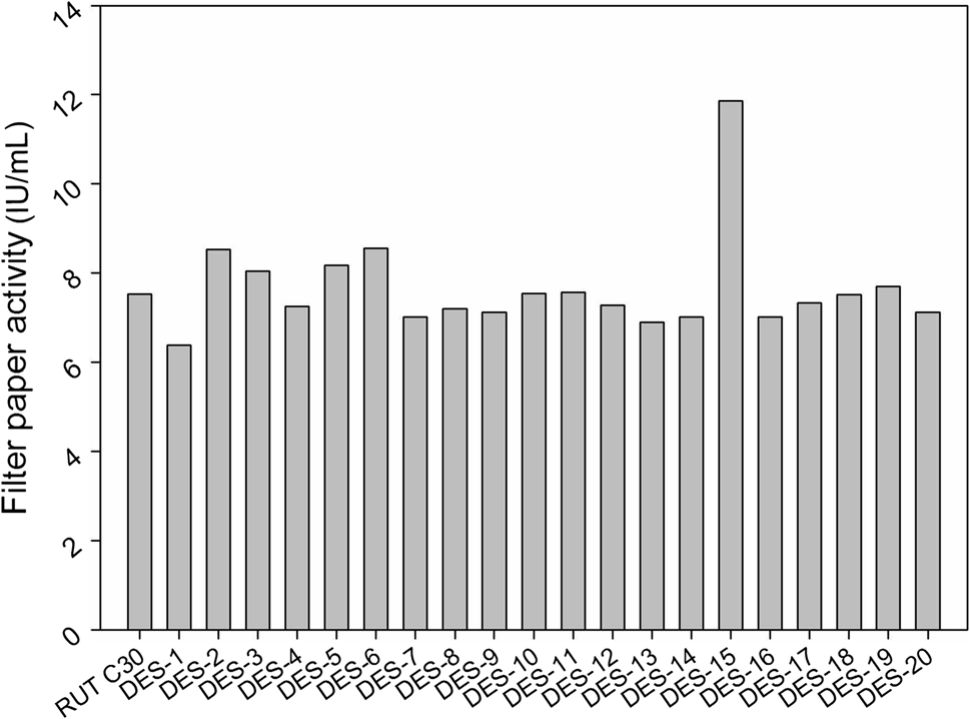
Screening of hyper-cellulolytic mutants. As this is an initial screening, data are representative for a single liquid fermentation only

Using an excellent cellulase producer RUT C30, 100 mutants were obtained by DES mutagenesis and inoculated into Erlenmeyer flasks for FPA determination. Among them, five mutants showed enhanced cellulase activities. Using a sample of 20 clones (Fig. 1), the cellulase activity for the parent strain RUT C30 was determined to be 7.11 FPU/mL, while the cellulase activity of one hyper-producing cellulase mutant, DES-15, was determined to be 11.86 FPU/mL, a 66 % increase in the overall cellulase activity. In contrast, however, the cellulase activities of the other four mutants were only marginally enhanced, increasing by 15–20 % when compared with the parent RUT C30. Given that DES-15 demonstrated the highest cellulase activity of the mutants, it was selected for further studies. The genetic stability of DES-15 was evaluated by determining the cellulase activity and secreted protein concentration in ten successive generations. The cellulase production and extracellular protein abundance of DES-15 were determined to be stable (Table S2).

**Fig. 2:**
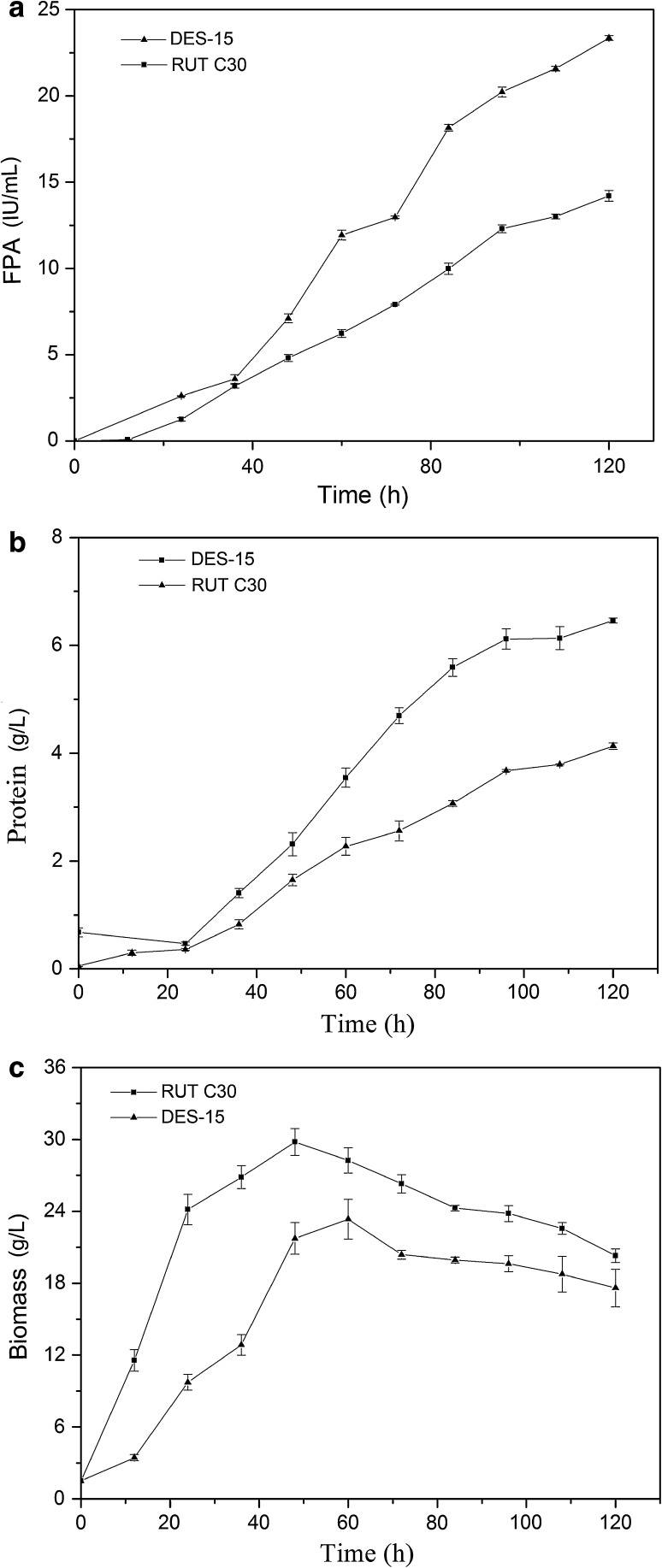
FPase activity (**a**), protein concentration (**b**), and biomass (**c**) in batch culture. The parent strain RUT C30 and the mutant DES-15 were cultivated in 5-L fermentor under conditions described in “Materials and methods”

Submerged cultivation in a fermentor is mostly used in the large-scale production of cellulase, as many parameters are easier to control in fermentors compared to flasks during large-scale production (Liu et al. 2013). To comprehensively compare the cellulase production of DES-15 with RUT C30, we first sought to determine the extracellular protein levels, biomass, and FPase activity during cultivation in a 5-L fermentor with the same cultivation parameters. As shown in Fig. 2a, the FPase activity of DES-15 was higher than that of RUT C30 throughout the fermentation process, with a final FPase of approximately 69 % higher activity being achieved by DES-15 when compared to the FPase of RUT C30. The protein concentration during the fermentation of DES-15 reached 6.5 g/L, which was nearly 50 % higher than that obtained by RUT C30 (Fig. 2b). Surprisingly, the biomass of DES-15 was lower than that of RUT C30 throughout the fermentation process (Fig. 2c). Biomass is considered to be one of the most important factors influencing productivity during submerged fermentation. However, although high productivity is generally achieved through increasing biomass, an overdose in biomass can limit the ability of oxygen to dissolve into the submerged cultures, resulting in impaired productivity (Olsvik and Kristiansen 1994; Wucherpfennig et al. 2010). In this study, we carefully controlled the influencing factors for all submerged cultures, demonstrating that the enhanced production of cellulase in DES-15 was independent of any variations in culture conditions. It is thus speculated that the enhanced production of cellulase in DES-15 might be due to an improved capacity for protein secretion, or a mutation leading to the increased transcription of cellulase genes as a result of the DES mutagenesis of RUT C30.

### The transcription abundance of the transcription factor *xyr1* and major cellulase genes are not altered in the cellulase hyper-producing mutant DES-15

**Fig. 3:**
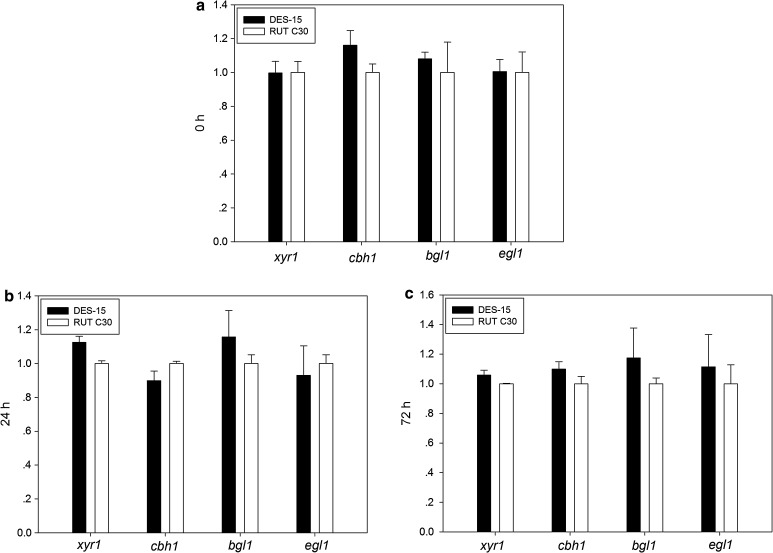
Expression levels of *xyr1*, *cbh1*, *bgl1*, and *egl1* in RUT C30 and DES-15 at 0 (before batch), 24, and 72 h (**a**–**c**). The *gpd1* gene was used for normalization, and the values were calculated by 2^−ΔΔCt^ methods. Experiments were performed in three independent replicates. *Error bars* represent the standard deviations. *Asterisks* were indicated significant differences at *P* < 0.05 according to Student’s *t* tests

In some cellulase hyper-producing strains of *T. reesei,* it was demonstrated that the course of mutagenesis caused an enhanced expression of the transcription factors and the genes related to cellulase production, responsible for the high cellulase production (Liu et al. 2013). Portnoy et al. (2011) assessed the transcription abundance of the transcription factor *xyr1* and the *cbh1* gene encoding cellobiohydrolase between the industrial hyper-producing strain, *T. reesei* CL847 and its obvious parent strain, RUT C30. The results showed that the expression of *xyr1* and *cbh1* was significantly higher in CL847 than it was in RUT C30. Among the known transcription factors, XYR1 is recognized as an essential transcription factor involved in cellulase/hemicellulase regulation in *T. reesei* (Mach-Aigner et al. 2008). To investigate whether the expression of *xyr1* was altered in the cellulase hyper-producing mutant DES-15 as a result of mutagenesis, qRT-PCR for the expression of *xyr1* was performed, and compared between the mutant DES-15 and the parent RUT C30 strain. As shown in Fig. 3a–c, the relative transcript abundance of *xyr1* was not significantly different in RUT C30 and its derivative DES-15 at any of the cultivation time points analyzed (0, 24, and 72 h). The expression levels of three other major cellulase genes *cbh1*, *bgl1*, and *egl1* were also measured by qRT-PCR. Again, no significant differences for any of the cellulase genes were observed between RUT C30 and DES-15, which were consistent with the equal expression levels of *xyr1* (Fig. 3a–c). These results suggest that the mutagenesis of RUT C30 did not affect the transcription abundance of the transcription factor *xyr1,* or any of the three major cellulase genes *cbh1*, *bgl1*, and *egl1*.

### Hyphal morphology of the cellulase hyper-producing mutant DES-15 in 5-L fermentor

**Fig. 4:**
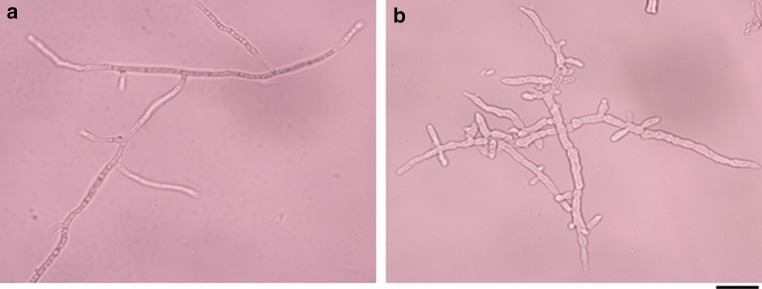
Hyphae morphology of the cellulase hyper-producing mutant DES-15 and the parent strain RUT C30 after 60 h cultivations. **a** RUT C30. **b** DES-15. *Bars* 20 μm

**Fig. 5:**
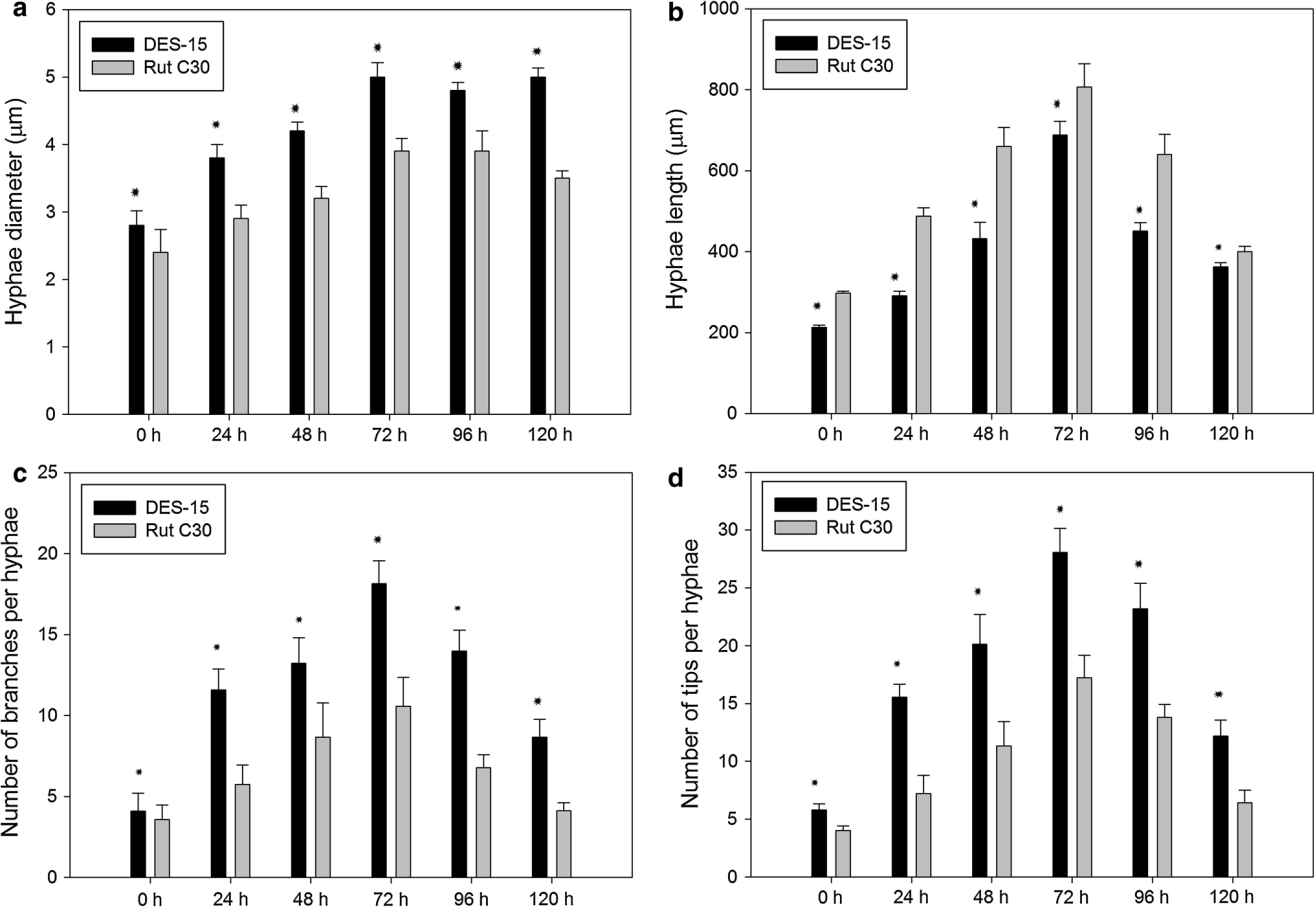
Hyphae characteristics of RUT C30 and DES-15 in batch culture at different culture time. **a** Hyphae diameter (μm). **b** Hyphae length (μm). **c** Number of branches per hyphae. **d** Number of tips per hyphae. More than 3000 hyphae were calculated from different view fields. Experiments were performed in three independent replicates. *Error bars* represent the standard deviations. *Asterisks* were indicated significant differences at *P* < 0.05 according to Student’s *t* tests

Having found that the transcript levels of the major cellulase genes and the transcription factor *xyr1* were not affected in DES-15, we investigated whether the protein secretion capacity of DES-15 was altered. It is generally believed that protein secretion in filamentous fungi mainly occurs at the tip of growing hyphae (Wösten et al. 1991; Xiang and Morris 1999), as extracellular proteins are more easily passed through the cell walls in the tips. Branched hyphae result in an increase in the number of tips, and thus enhance the secretion of proteins into the fermentation broth (Wessels 1993). Therefore, it is speculated that the hyphal morphology, especially branching of DES-15, may have been altered in the submerged cultures. As shown in Fig. 4a, the hyphae in the parent strain RUT C30 were elongated, and not highly branched. However, in contrast, the hyphae of the mutant DES-15 were shorter and more branched than those of their parent strain RUT C30 (Fig. 4b). To accurately analyze the differences in hyphal morphology between DES-15 and RUT C30, their hyphal growth parameters were measured over time. Such measured parameters included the numbers of branches, numbers of tips, hyphae length, and hyphae diameter as seen in the 5-L fermentor. Figure 5c, d shows that the DES-15 mutant has a greater mean number of branches and tips per hypha across the fermentation period. In addition, a larger hyphae diameter could be measured for DES-15 than the parent RUT C30 (Fig. 5a), while the hyphae length was significantly longer in the RUT C30 parent strain than in the DES-15 mutant (Fig. 5b). In *T. reesei*, the hyphae morphology, particularly the number of hyphae tips, can be altered by many factors, such as agitation and culture medium composition (Papagianni 2004; Ahamed and Vermette 2009, 2010). In this study, the mean number of tips per hypha in the DES-15 mutant was significantly greater than those seen for the RUT C30, largely due to DES-15′s highly branched hyphae that were observed throughout the fermentation process. The results revealed that the mutagenesis of RUT C30 led to the significant changes in the morphology of DES-15, specifically with regard to branching. These changes likely contribute to the enhanced protein secretion capacity of DES-15 seen during submerged cultivation.

### Effect of highly branching hyphae on protein secretion

**Fig. 6:**
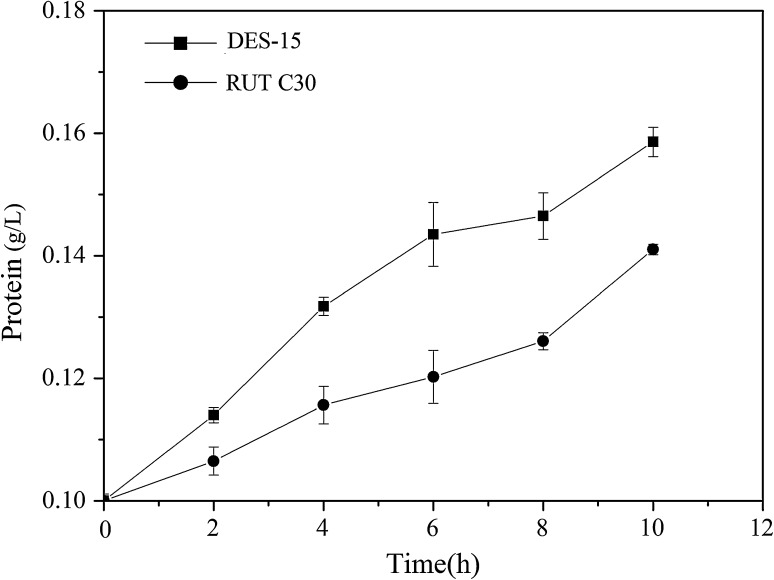
Protein concentration of RUT C30 and DES-15 with the same biomass. The inoculums with the same biomass were inoculated in fermentation media. Protein concentration was measured every 2 h

Having demonstrated that DES-15 had a high capacity of protein secretion despite having a lower biomass compared to RUT C30, we sought to elucidate how these alterations to the branching of the hyphae affected protein secretion. To explore this question, the protein concentration of the same biomass of DES-15 and RUT C30 was determined. Inoculums with the same biomass were inoculated in the fermentation medium, and the protein concentration was measured every 2 h until the biomass differed. As shown in Fig. 6, the protein concentration of DES-15 was much higher than that of RUT C30 during the whole fermentation process, demonstrating that DES-15 possessed a higher protein secretion capacity than RUT C30. The results suggested that the cellulase production increase was due to the differential hyphal morphology of DES-15, and that this strain is thus particularly suitable for large-scale cellulase fermentation.

### Transcriptional analysis of the genes involved in hyphal branching

**Fig. 7:**
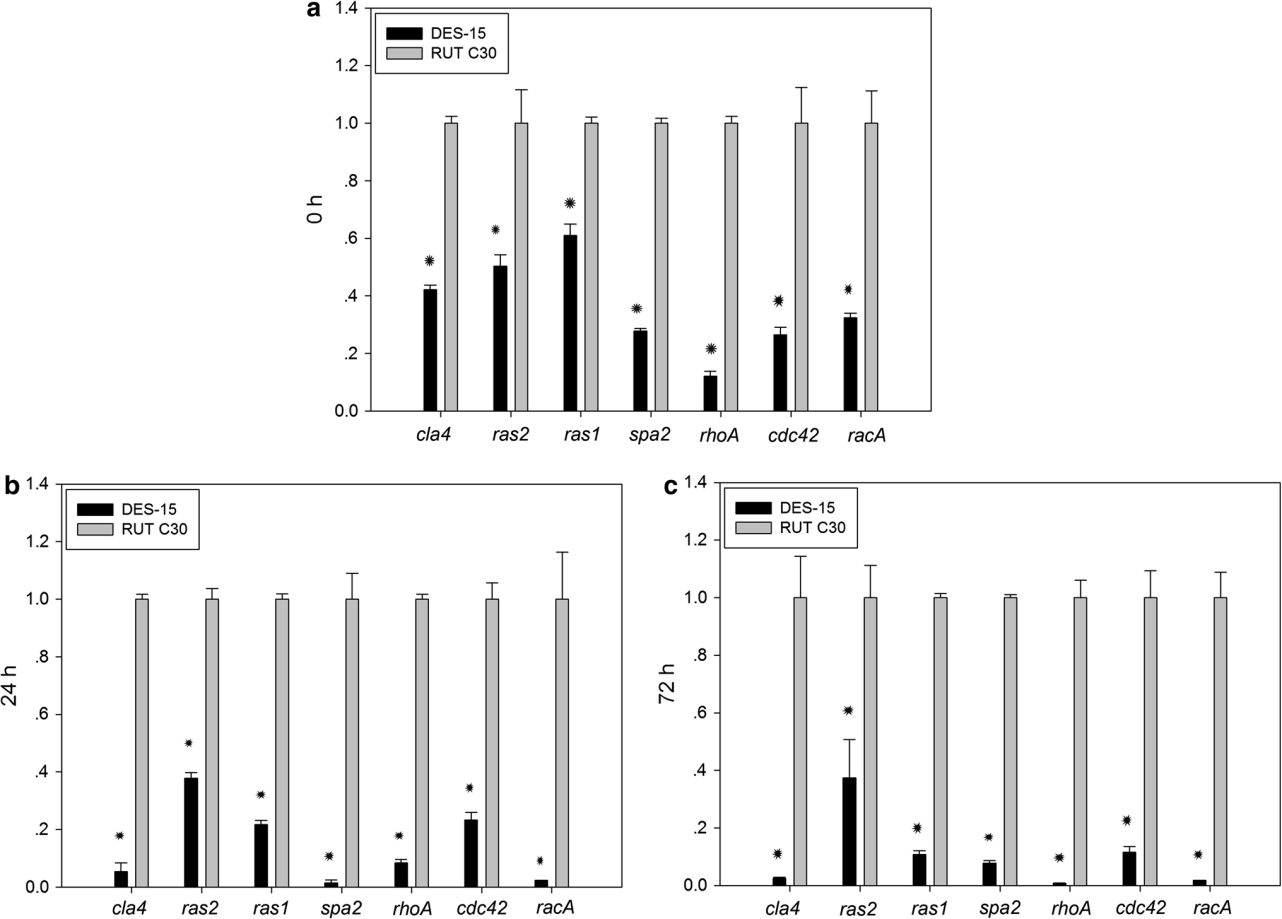
Expression levels of seven genes (*cla4*, *spa2*, *ras2*, *ras1*, *sepa*, *cdc42,* and *pxl*) involved in branching in RUT C30 and DES-15. (**a**–**c**) Transcription abundances of *cla4*, *ras2*, *ras1*, *spa2*, *rhoA*, *cdc42,* and *racA*. Mycelia were collected at 0 (before batch), 24, and 72 h. The *gpd1* gene was used for normalization, and the values were calculated by 2^−ΔΔCt^ methods. Experiments were performed in three independent replicates. *Error bars* represent standard deviations. *Asterisks* were indicated significant differences at *P* < 0.05 according to Student’s *t* tests

It has been previously reported that several genes are involved in hyphal branching in filamentous fungi (Harris 2008). To identify the reasons for the highly branched morphology seen in DES-15 mutant, the expression levels of seven genes previously reported to be involved in branching (Cla4 protein ID 71315, RhoA protein ID 119871, Spa2 protein ID 108829, RacA protein ID 47055, Ras1 protein ID 120150, Ras2 protein ID 110960, and Cdc42 protein ID 50335) were examined by qRT-PCR at different cultivation times (Kwon et al. 2013). When comparing the initial time of cultivation (before batch, Fig. 7a) to that at 24 h post-cultivation (Fig. 7b), the relative transcript abundances of *cla4*, *ras2*, *ras1*, *spa2*, *rhoA*, *cdc42,* and *racA* were significantly decreased in the DES-15 mutant when compared with the parent strain RUT C30. After 72 h, transcript abundances of the seven genes relating to branching were shown to decrease further in DES-15, and the differences in expression between DES-15 and RUT C30 were significantly greater (Fig. 6c), suggesting that the decreased expression of these seven genes might contribute to highly branched hyphae in DES-15.

Both the extracellular environment and intercellular processes influence hyphal branching. Reported mutants with branching-specific phenotypes have suggested that branching formation does not involve a simple recapitulation of an established branching mechanism (Harris 2008). Only a few genes have thus far been reported to influence branching in filamentous fungi. In *Ashabya gossypii*, *Agcla4* is required for the maturation of young hyphae. The development pattern of *ΔAgcla4* was shown to be very similar to that of the wild type, apart from a slight increase in lateral branching (Ayad-Durieux et al. 2000). Mutations in *AgSPA2* have an effect on the hyphal tip growth speed and the overall branching density. Moreover, the apparent hyper-branching phenotype can be observed in the null mutants of *AgSPA2* (Knechtle et al. 2003). In addition, some members of GTPase proteins, such as RhoA, Ras1, Ras2, RacA, and Cdc42, play key roles in hyphae growth, especially with regard to hyphal branching in filamentous fungi. RhoA plays a role in the emergence of the secondary germ tubes and branches in *Aspergillus nidulans*. Two different mutants *rhoA* ^G14V^ and *rhoA* ^E40I^ obtained from the site directed mutagenesis and performed different phenotypes. The emergence of lateral branches in the *rhoA* ^G14V^ strain was delayed and branches were clustered, while in the *rhoA* ^E40I^, the emergence of secondary and tertiary germ tubes and branches was accelerated, and lateral branches emerged in irregular numbers (Guest et al. 2004). In *T. reesei*, the *ΔTrRas1* strain exhibited highly branched, swollen hyphal cells, in contrast to its parent strain TU-6. The *ΔTrRas2* mutant formed aggregated hyphae displaying a hyper-branching phenotype (Zhang et al. 2012). The same phenomenon was also found in the null mutant of Ras2/RasB in *N. crassa* and *A. fumigatus* (Momany 2002). In the important biological control agent *Nomuraea rileyi*, the hyphae of the RNAi-silenced mutant *racA*RM were largely thicker with increased apical branching, whereas those of the RNAi-silenced mutant *cdc42*RM did not exhibit any observable differences, except for an increase in the number of branches when compared with the wild type strains (Jiang et al. 2014). It can, therefore, be concluded that these genes have the potential to negatively impact hyphal branching. These observations indicate that the decreased expression of these genes in DES-15 may contribute to the highly branched hyphae seen here, which is consistent with the results of previous studies.

## Conclusions

This work focused on the effect of high-branching hyphae on cellulase production in a cellulase hyper-producing strain DES-15 that was obtained through the DES mutagenesis of the parent *T. reesei* strain RUT C30. In batch cultures, the hyphae of DES-15 were more highly branched and had a larger number of actively growing tips than those of its parent strain RUT C30, allowing for better protein secretion. The expression of genes thought to negatively regulate branching was shown to be significantly lower in DES-15 than in RUT C30. This study reveals a direct relationship between fungal morphology and overall cellulase production by *T. reesei* DES-15.

## Electronic supplementary material

Below is the link to the electronic supplementary material.

Supplementary material 1 (DOCX 15 kb)
