# Supplementary material for: Effect of highly branched hyphal morphology on the enhanced production of cellulase in Trichoderma reesei DES-15
Source: 3 Biotech. 2016 Oct 5;6(2):214. doi: 10.1007/s13205-016-0516-5 (PMC5052222; doi:10.1007/s13205-016-0516-5)
Supplement: Supplementary file 1 — Supplementary material 1 (DOCX 15 kb) [file 13205_2016_516_MOESM1_ESM.docx]

**Tables**

**Table S1 Primers used in this study**

| Name | Sequence (5’-3’) |
| --- | --- |
| gpd1F  gpd1R  qRas1F  qRas1R  qRas2F  qRas2R  qCla4F  qCla4R  qRhoAF  qRhoAR  qCdc42F | GGAGCTCTTTGAAGAGGA  GGCAGGTACTTGACGTTTTC  GGGTTTCTTGCTGGTCTACTC  TGTCCTTGACTCTCAGGATCT  ACGGTGGTGTCGGTAAGA  GCGGTATGAGTCCTCAATTGTC  CATCGACTTCCACAAGACAGAG  CGCTTGATCTCGAAGATGGT  CGGCTGGACAGGAAGATTATG  CAGAGAGTCAGGAGAGTCGATAG  CCAACAAGTTCCCTTCGGAATA |
| qCdc42R | TCGAACAGTCCCAGGGTATAA |
| qRacAF | GCAAGACCTGTCTTCTCATCTC |
| qRacAR | GGCTTGCCATCAACAATGAC |
| qSpa2F | CATGAGCATGAATGGCTTCC |
| qSpa2R | CGTACATGTTCATGCCGTTG |

**Table S2 Genetic stability of DES-15**

|  | **Protein (g/l)** | **FPA (IU/ml)** |
| --- | --- | --- |
| F1 | 3.45±0.64 | 11.86±0.76 |
| F2 | 3.52±0.39 | 11.95±0.79 |
| F3 | 3.41±0.45 | 11.74±1.11 |
| F4 | 3.55±0.10 | 11.98±0.58 |
| F5 | 3.37±0.41 | 11.76±0.98 |
| F6 | 3.62±0.28 | 12.04±0.42 |
| F7 | 3.42±0.30 | 11.85±0.71 |
| F8 | 3.43±0.27 | 11.80±1.06 |
| F9 | 3.47±0.11 | 11.91±0.87 |
| F10 | 3.45±0.17 | 11.83±0.21 |
